# Review Mechanisms for Advanced Medical Therapies in Japan and Thailand: A Proposal for the Use of Expert Clinical Benefit Assessments at Designated Institutions

**DOI:** 10.1007/s41649-024-00301-9

**Published:** 2024-08-15

**Authors:** Kenji Matsui, Nipan Israsena, Jaranit Kaewkungwal, Pornpimon Adams, David Wendler, Reidar K. Lie

**Affiliations:** 1 Division of Bioethics and Healthcare Law, Institute for Cancer Control, National Cancer Center Japan, Tsukiji 5-1-1, Chuo-Ku, Tokyo, 104-0045 Japan; 2 Center of Excellence for Stem Cell and Cell Therapy, Faculty of Medicine, Chulalongkorn University, 8th Floor Bhumisiri Bld ., Rajdamir Rd, Lumpini, Pathumwan, Bangkok, 10330 Thailand; 3 Department of Tropical Hygiene, Faculty of Tropical Medicine, Mahidol University, 420/6 Rachavithi Rd, Rachthewi, Bangkok, 10400 Thailand; 4 Office of Research Services, Faculty of Tropical Medicine, Mahidol University, 420/6 Rachavithi Rd, Rachthewi, Bangkok, 10400 Thailand; 5 Department of Bioethics, National Institutes of Health Clinical Center, Bldg. 10, Room 1C118, Bethesda, MD 20892-1156 USA; 6 Department of Philosophy, University of Bergen, Sydnesplassen 12, 5007 Bergen, Norway

**Keywords:** Advanced new therapies, Scientific and ethics review, Stem cell regulation, Regenerative medicine

## Abstract

Advanced new therapies, such as stem cell and gene therapies and xenotransplantation, represent challenges for regulatory and ethical review. Major drug agencies, such as in the U.S., India, and Europe, have asserted regulatory authority and require ethics review by local ethics review committees, using the same strict requirements as those for standard drug approvals. In spite of this, unapproved and undocumented stem cell clinics flourish in all of these places, suggesting that current approaches do not offer patients sufficient protection. Japan has attempted another approach, requiring approvals at local levels for all regenerative medical procedures, and a faster approval of promising new interventions. The Japanese approach has, however, also been criticized as not striking a proper balance between early access and a proper assessment of safety and effectiveness. For smaller and less-resourced countries, such as Thailand, one major challenge is limited expertise to conduct the evaluation of these advanced new therapies. This article provides an overview of the issues facing regulators and proposes that countries should restrict the early adoption of advanced new therapies to specialized clinics with appropriate scientific and ethical expertise for review. Review in these institutions should focus on expert clinical benefit assessments for individual patients being offered such interventions, independently of whether they are offered as research or therapy.

## Introduction

Since the 1960s, a rigorous approval process for drugs has been adopted by most countries, requiring approval by national drug regulatory authorities before any use, irrespective of whether the use is for therapy or research. Marketing authorization for clinical use generally requires positive results from formal clinical trials. Emerging technologies, such as cell- and tissue-based interventions, gene therapies, and xenotransplantation are different from traditional drugs in that they are more complex interventions than a standardized administration of a new chemical entity. In that respect, they are more like surgical interventions that have not generally required approvals by national regulatory agencies. Nevertheless, major drug regulatory agencies have asserted that they are also authorized to regulate these emerging technologies. For example, the two recent cases of transplantation of a pig’s heart in Baltimore could only be done after the U.S. Food and Drug Administration (FDA) had authorized it for those specific patients under their compassionate use rules.

In addition to national approval by drug regulatory agencies, drugs and other interventions used for human subjects research require local or regional research ethics review approval. Many countries therefore have a dual review system, requiring approval both at the national and local levels. However, as we shall argue in this paper, this dual system is still not capable of regulating emerging technologies appropriately, both in rich and emerging economies.

In this paper, we shall propose that we need a different review mechanism focusing on Expert Clinical Benefit Assessments of these new interventions. Such reviews can best be done at selected institutions designated by national governments for this task. This is particularly important for emerging economies which often have limited expertise, even at the national level. A recent White Paper produced for the World Economic Forum in 2022 emphasizes that gene therapies promise to address major health problems in low- and middle-income countries, but that effective research and development faces a number of challenges, such as a lack of expertise, infrastructure and regulatory capacity (World Economic Forum 2022). In a case study from Thailand on the development of a gene therapy product for beta thalassemia, affecting between 3 and 9% of newborns in Thailand, inadequate national regulations were identified as a major issue (World Economic Forum 2022), although the Thai FDA is aligning with major regulators overseas, in the U.S., Europe, Japan, and Australia. However, even with the alignment of criteria for approval of products and procedures, there are still complex judgments to be made about the suitability of patients for early use, both in research and therapy, of these complex interventions. It is not clear whether local research ethics review committees, or clinical ethics committees, can deal properly with these types of cases. Similar issues are raised for other low- and middle-income countries.

We shall first present the challenges currently faced by national regulatory authorities when evaluating emerging technologies. We shall use the term *emerging technologies* to identify what are commonly referred to as cell- and tissue-based interventions or advanced therapies. We shall argue that the model adopted in the U.S., E.U., and India, where the national drug regulatory agency has the authority to evaluate and approve emerging technologies, is not sufficient. Moreover, we shall also argue that the more decentralized approach taken by Japan will not be appropriate. We then conclude by proposing a policy option that may offer a better way to address the regulation of emerging technologies, particularly for lower and middle income countries.

## National Regulations by Existing Drug Regulatory Authorities

In the West, major national drug regulatory agencies have asserted regulatory authority for some or all of these technologies. In the U.S., for example, cell- and tissue-based products, including stem cells, gene therapies, and xenotransplantation are regulated by the U.S. FDA in the same way as new chemical entities. The FDA’s Center for Biologics Evaluation and Research is responsible for the approval process. New cell- and tissue-based products can be administered in clinical research only after approval by the U.S. FDA. Market authorization requires the same standard of evidence (i.e., proper clinical trial evidence) as has traditionally been required for authorization of standard drugs. In Europe, the European Medicines Agency (EMA) has adopted similar rules, under their regulations for *Advanced therapy medicinal products* (ATMP). These include gene therapies, somatic-cell therapies, and tissue-engineered medicines. Similarly, in India, drug regulations were revised in 2019 and now explicitly state that stem cell–derived products, gene therapy products, and xenografts need to follow the same approval procedures as regular drugs, i.e., approvals are needed for research and marketing.

However, there are important differences in what products are excluded. Under the U.S. regulations, minimally processed tissue is defined narrowly, and only includes procedures that do not alter the basic composition or function of the tissue, and is subsequently given to an individual to perform the same function as the original tissue. It excludes the separation of cells, as is done to produce stromal vascular fraction from fat tissue, and also the return of minimally processed tissue to perform other functions, such as the return of fat cells to influence neurological function. In India, there is a wider definition of minimal manipulation, which could include tissue that is intended to perform a variety of biological functions in the receiving individuals. The Indian Council of Medical Research (ICMR) and the Department of Biotechnology (DBT) at the Ministry of Health and Family Welfare issued joint guidelines in 2017 and updated reviews specifying which interventions they have endorsed (Indian Council of Medical Research & Department of Biotechnology 2017). For stem cells - similar to the situation in the U.S. - they have, to date, endorsed only bone marrow transplantation and one treatment for an ocular disease.

The approaches taken by these three regions/countries are in line with a general consensus, as reflected by WHO guidance documents and guidelines developed by the *International Society for Stem Cell Research* (ISSCR) (World Health Organization 2023b; Daley et al. 2016; World Health Organization 2022). New interventions should be approved based on rigorous evidence, and until such approval, they should only be available under approved research protocols. Such research use also needs to be approved by the relevant regulatory authority, who, at least in the early stages of the research, will mostly be concerned with the safety of the interventions and whether the proposed research is likely to provide reliable evidence to support clinical approval of the interventions. In addition, research use will also be reviewed by regular research ethics committees (RECs) or institutional review boards (IRBs), and sometimes by specialized committees set up to review stem cell or gene therapy research. In principle, therefore, the review and approval process for these emerging technologies can rely on the general review structure adopted in most countries since the 1960s.

In spite of the relatively strict national regulations, clinics that provide unapproved stem cell treatments are flourishing in all three countries/regions. In 2016, there were 570 clinics operating in the U.S., and by 2021, this had increased to almost 1500 businesses operating 2754 stem cell clinics (Turner 2021). This is in spite of attempts by the U.S. FDA to close unlicensed stem cell clinics. The situation in the U.S. demonstrates the difficulties of enforcement, even for an agency as powerful, and well-staffed, as the U.S. FDA. It highlights the influence of powerful interests to provide undocumented but promising interventions to patients. The EMA issued a notification expressing their concern about the growth of stem cell clinics in Europe providing undocumented interventions (European Medicines Agency 2020).

However, in India, neither ICMR nor DBT have any general regulatory or enforcement authority over clinical activities in clinics and hospitals; they can only issue guidelines or recommendations. The legally binding regulations do appear to allow stem cell interventions for clinical use that the guidelines specify can only be labeled as clinical research.

The growth of clinics offering undocumented interventions for serious conditions, often at high prices, illustrates the pressure on regulatory authorities to approve emerging technologies quickly. Agencies such as the U.S. FDA have over the years introduced flexibilities, such as rules governing compassionate use, expanded access, accelerated approval programs, and emergency approval. The approval for the transplant of a pig heart in Baltimore was based on compassionate use rules, for example; the U.S. FDA did not give approval for this procedure in a general research project that would systematically recruit new patients. In general, the criteria for approval seem to have been relaxed, releasing interventions for general use with less evidence for their effectiveness than has been required earlier, due to a large degree of political pressure and pressure from patient advocacy groups, who are at least partially funded by the pharmaceutical industry. In 2021, for example, the U.S. FDA approved an Alzheimer’s drug, Adhuhelm, in spite of the recommendation against such approval by the expert committee, leading to the resignation of several committee members (Maulden 2022; Lewis-Kraus 2023). The accelerated approval program for cancer drugs has similarly been criticized for approving drugs based on insufficient data, and not following up on accelerated approvals appropriately (Gyawali et al. 2021; Gyawali et al. 2023).

Requiring national approvals for emerging technologies has clearly not been sufficient to deter the introduction of interventions that have not undergone rigorous review. Japan’s regulatory framework for regenerative medicine is often portrayed as an alternative model legislation, effectively balancing the protection of patients and promoting rapid access to promising, new interventions. It is to a consideration of this approach that we now turn. 

## The Regulatory Framework for Regenerative Medicine in Japan

The current framework in Japan was introduced in 2014 by the passing of the *Act on the Safety of Regenerative Medicine* (ASRM). There are three important features of this Act.

First, regenerative medical interventions are divided into three risk categories (Tobita et al. 2016). Class I products are those that have been rarely used in humans and involve unknown effects on human life and health, or potentially serious effects (e.g., gene-modified cells/ex vivo gene therapy products; xenogeneic cells). All stem cell products derived from sources other than the individual patient receiving the cells are included in this category.

Class II products are those that have been used in humans and pose intermediate risk (e.g., cultured autologous stem cells or uncultured but heterologously used autogenic stem cells). This group includes cells obtained from the recipients but manipulated in some way, such as augmentation by culture. This class also includes cells that are used for a different purpose, for example, using stem cells isolated from mesenchymal tissue to repair the patient’s atherosclerotic arteries. Class III products are those that have been minimally manipulated and utilize the function of the somatic cells of origin and, therefore, are considered low risk (e.g., uncultured and homologously used autologous stem cells).

Second, ASRM has a single regulatory framework which includes a “therapy track” and a “research track”. On either track, a physician/researcher must prepare a clinical/research protocol, undergo a review by the ASRM-designated committee (described below), and submit the approved protocol to the Minister of the Ministry of Health, Labour and Welfare (MHLW) before providing a regenerative medicine intervention. The framework is therefore comprehensive, requiring explicit approval procedures for all regenerative medicine products and services.

Third, there are ASRM-designated committees for the evaluation of these products both at the institutional and national levels. At the institutional level, there is a requirement for review of Class I and II products by a *Certified Special Committee for Regenerative Medicine* (CSCRM). These committees are accredited by the MHLW as possessing sophisticated review ability and independence. Class I products also need to be reviewed by the *Health Sciences Council Regenerative Medicine Evaluation Expert Committee* at the national level.

As Class III products involve lower risk than Class I/II, the protocol with Class III products can be reviewed by the Certified Committee for Regenerative Medicine (CCRM, which has less stringent membership and expertise requirements than the CSCRM). The MHLW provides two templates for protocols: one for those on the therapy track and the other for those on the research track. In either case, the application will be reviewed by the same committee.

The reported number of protocols with regenerative medicine on the “therapy track” was 7 at Class I, 1500 at Class II, and 3868 at Class III, while the number on the “research track” was 17 at Class I, 44 at Class II, and 48 at Class III (as of the end of October 2023) (Ministry of Health, Labour and Welfare of Japan 2023). Except for those with Class I products, the overwhelming majority of regenerative medicine interventions in Japan are therefore implemented in the less demanding “therapy track” (Ikka et al. 2023). The therapy track permits approval for general, clinical use based only on a plausible case that the intervention might benefit patients, without any requirement to assess the intervention in research. In contrast, the research track is very similar to what a typical research ethics committee would require. In particular, it requires documentation of a proper scientific protocol and research demonstrating safety.

The other important element in the Japanese regulation of stem cells is the relaxation of the evidence required for regenerative medicine products to be used as approved therapeutic interventions. The *Pharmaceutical Affairs Law* was amended in 2014 to include separate chapters for drugs, devices, and regenerative medicine products and renamed the *Pharmaceutical and Medical Device Act*. This allows conditional approval of cell-based products, based on significantly more limited evidence than full-scale phase III trials.

For serious diseases with no satisfactory treatments, an intervention can be approved with more limited evidence than usually required for drug approval. As an example, *HeartSheet* involves the implantation of a cell sheet of stem cells from the patient’s own skeletal muscle cells and would fall into Class I, subject to stringent national approval. However, it was given conditional approval in 2015 to treat heart failure as a result of ischemic heart disease (Editorial 2015) based on data from a clinical trial with seven patients and supporting data from 19 patients with related heart conditions.

Conditional approval requires that the sponsor continues to gather evidence for the safety and effectiveness of the intervention with the aim of gaining full approval within a 7-year period. There are also requirements that patients who receive conditionally approved interventions are fully informed of the status of the intervention. However, there is no additional requirement for a formal REC/IRB review, as the initial ASRM-mandated review takes the place of a formal REC/IRB review.

The original approval for *HeartSheet* came with the condition that “within 5 years, the company would have to provide data from at least 60 patients treated with *HeartSheet* and 120 controls to show that the treatment is effective” (Editorial 2015). Halfway through this period, fewer than ten patients had been recruited (Editorial 2018). In November 2018, it was agreed that the conditional approval deadline would be extended by 3 years and was scheduled to be reset to the end of September 2023 (Kubota 2022). Reportedly at the time of writing, the company has applied for full approval by this set deadline, but no formal approval had been granted as of May 2024 (Imamitsu 2024). It should also be noted that, since patients at least partly pay for their expenses when receiving this intervention, it essentially involves patients subsidizing the company’s costs of developing a new intervention.

A clinical trial by the same group was approved in 2018, now using induced pluripotent stem cells derived from heart muscle cells (Cyranoski 2018). The condition is: once this trial has recruited around ten patients and is found to be safe and effective in these patients, the company can again apply for conditional approval. On May 19, 2023, the research team held a press conference, reporting that eight patients in total were transplanted and the trial was completed. They said that they would submit an application to the Pharmaceuticals and Medical Devices Agency (PMDA) to re-obtain a conditional time-limited approval for *HeartSheet*, expecting the realization of its clinical application within two years (The Science News 2023).

Even those who are sympathetic to the U.S. FDA approach have pointed out that the FDA often lacks sufficient capacity to effectively monitor and enforce compliance. Proponents of the Japanese system argue that it is better to have an imperfect, universal requirement of review and approval by designated local committees. This approach, they argue, provides a review of all new interventions without unnecessarily delaying access to promising interventions for serious illnesses that have no existing satisfactory treatments. Critics, however, point to several problems and challenges with the Japanese approach.

In a series of news articles and editorials, the journal *Nature* has strongly criticized the Japanese approach. They point out that treatments approved through the Japanese therapy track are routinely offered to patients without evidence of their effectiveness, often at high prices. One example is the injection of stem cells derived from fat for the treatment of amyotrophic lateral sclerosis (Cyranoski 2019).

Once therapies are approved for finance through the health insurance system based on preliminary data, there is little incentive for the sponsors to produce the required higher-quality data. In particular, since “promising” treatments are available, and reimbursed, there are no incentives for patients either to enter proper randomized controlled clinical trials.

Recently, a commission in Japan found that the review procedures for regenerative medicine have serious deficiencies. Protocols were approved that did not provide proper references to data documenting effects and had clinicians or researchers in charge who did not have the appropriate expertise for the diseases that were being treated (Ikka et al. 2023). Clearly, the regulatory framework in Japan has also not achieved an appropriate balance between providing access to therapies that are likely to benefit current patients with obtaining adequate evidence about safety and a degree of efficacy prior to widespread adoption.

From this brief review of the policies and practices in various countries, it is clear that innovative and emerging technologies, such as cell-based interventions, represent challenges for the regulatory systems. Strict national regulations, with the possibility of sanctions, as in the U.S., as well as national rules and guidelines, with requirements for local research ethics review, as in India, have not prevented interventions from being offered by clinicians without proper evaluations of their potential clinical benefits. It is clear that emerging technologies present challenges for wealthy countries, such as the US and Japan, but also for large, emerging economies such as India. In the next section, we shall describe additional issues facing smaller, emerging economies, using Thailand as an example.

## The Drug Regulatory Framework in Thailand

The U.S., E.U., and India all have well-developed national drug regulatory systems that have been used as a framework for regulating emerging technologies. Thailand is a country with a similarly classified national drug regulatory system but has not yet introduced national rules for emerging technologies (World Health Organization 2023a).

In Thailand, the primary government agencies responsible for regulating clinical care are the three branches of the Ministry of Health: the Thai FDA, which regulates medical products registration; the Department of Health Service Support (DHSS), which monitors and sets standards for hospitals and clinics in the country; and the Medical Council of Thailand (TMC), which controls standards of practice of physicians.

There are no laws that directly regulate research involving human subjects. Instead, research involving human subjects is the responsibility of the host institution and its local REC/IRB. Nevertheless, the Thai FDA considers data only from research conducted with the approval of a limited number of RECs/IRBs that have been certified by the Thai FDA to oversee research to be used as the basis for marketing approval of products. In addition, the TMC requires that all physicians practicing in Thailand follow specific guidelines when they conduct clinical research.

The limitations of this long-existing system became evident in 2008–2010. At the time, there was widespread public misunderstanding about stem cell treatment for incurable diseases, leading to an increasing number of doctors and clinics offering patients so-called novel stem cell treatments. Since there were no clear rules set by the TMC (e.g., treatments need to be evidence-based), many private doctors considered various types of cell injection a medical care procedure, not research. As a result, there was no need for permission from a REC/IRB, nor approval by the Thai FDA. On the other hand, many clinical research protocols using stem cells received approval from local RECs/IRBs with insufficient supporting scientific and safety data, poor study design, misleading patient information sheets, and deficient informed consent. Several factors contributed to discrepancies in REC/IRB standards for research involving stem cells, including different levels of training, a limited number of stem cell experts in the country, unclear regulation, and conflicts of interest. These shortcomings reflect the need for a central unbiased board with sufficient expertise to review treatments involving advanced technology.

To mitigate this problem without drafting a new law, the TMC revised the rule of practice for doctors who perform stem cell treatment and clinical research involving stem cells in Thailand. Similar to the guidelines published by the ICMR and DBT in India, the critical message was that the only stem cell treatment that is approved for treatment in Thailand is hematopoietic stem cells used for hematologic conditions.

Until the TMC officially recognizes other stem cell treatments, every procedure that administers stem cells inside the human body is considered research and requires approval from one of the RECs/IRBs recognized by the TMC, which are primarily located in large medical schools and the Ministry of Health. These proposals must also get approval from the newly formed TMC scientific and ethical committee for stem cell research. Doctors who do not follow the rules may get their licenses revoked.

The stem cell regulation rule became effective in 2011. New stem cell treatments approved as standard therapies in Thailand required submission of supporting data to the TMC or to a professional organization recognized by the TMC. Since then, by 2021, only cultivated corneal epithelial stem cells for limbal stem cell deficiency have been approved as standard stem cell therapy in the country by and can be provided as a service according to the guidelines set by the TMC and Royal Society of Ophthalmology. The approved therapy is qualified to be submitted for potential reimbursement support from the national coverage policy.

In Thailand, since the review of new advanced therapies is basically the responsibility of local RECs/IRBs, the growing list of innovations, such as chimeric antigen receptor T-cell (CAR-T) therapy, gene therapy, genome editing, pluripotent stem cell–derived cells, and personalized cancer vaccines has made it challenging for regular RECs/IRBs to evaluate projects effectively. This is particularly so because evaluating each technology requires input from specialized, independent experts, which is challenging to find in a country such as Thailand. Other issues previously encountered with stem cell treatments, like public misperception, conflict of interest, and widespread misinformation, apply as well. Without a clear regulatory pathway, some doctors argue that applying these technologies to a single patient is clinical care or compassionate use, not research; therefore, it does not require REC/IRB approval. And vulnerable patients are easily tempted to pay a high price for ineffective and potentially harmful procedures.

The Thai Ministry of Health drafted a new law called the *Cell Therapy Act* to strengthen the legal authority of all regulating agencies. Under the drafted law, all physicians and centers that provide advanced therapy must register for approval and monitoring by TMC and DHSS; failure to comply and to be involved in illegal advertisement can lead to jail time. The drafted law also creates a central board for regulating treatment using advanced technology. Although this draft law passed the public hearing process in 2019, at the time of writing (2024) it is still unknown when or whether it will become effective.

While the strict regulatory systems and guidelines in the U.S., E.U., India, and Thailand probably have reduced malpractice and substandard human research involving stem cells, the persistent presence of the widespread use of unapproved interventions demonstrates that there is continued clinical demand for them. Also, in Thailand and India, the apparent reluctance to introduce legally enforceable regulations shows that there is considerable resistance among relevant professional and patient groups, reflected also in the pressure in the U.S. to approve new interventions more quickly. It is therefore unlikely for a country such as Thailand to expect that introducing a national requirement for regulatory review of emerging technologies will solve the problem of the use of unproven interventions by medical practitioners. In the E.U., U.S., and India, it is possible to identify a sufficient number of independent experts for national review, which would not be possible in smaller lower- and middle-income countries. This is in addition to the challenge of enforceability in the context of enormous pressure to make these interventions available to patient groups, professionals, and industries.

## Towards Expert Clinical Benefit Assessment (ECBA): A Novel Approach

We have identified several challenges for the review and approval of emerging technologies. Even well-resourced national regulatory authorities cannot prevent the widespread use of undocumented interventions in clinics. There is pressure from patient groups to approve quickly promising interventions, even before sufficient evidence of effectiveness has been obtained. Early use of even approved interventions often involves complex individual risk–benefit evaluations, in addition to the trade-off between benefits for current against future patients. Existing local research ethics review committees often do not have the expertise to make such judgments appropriately nor do all institutions have the expertise to conduct serious clinical research and use promising interventions appropriately. This was noted over a decade ago for stem cell interventions but applies in general to the whole class of advanced therapies (Taylor 2010). The Japanese solution was to move the review responsibility to local committees especially designated for the review of specific new interventions, but this also does not solve the problem, unless one can ensure that the local committees have the necessary expertise and can carry out a competent review. However, in spite of the general deficiencies in the review system documented by the government evaluation committee in Japan, there are also review committees that do a thorough evaluation of new proposals and require them to go through the research track with proper protocols satisfying standard scientific criteria. What is required, therefore, is a new type of review, which we will call Expert Clinical Benefit Assessment (ECBA) before specific emerging technologies are adopted. Such review will follow the general safety and potential benefit assessment conducted by national regulatory authorities, and also the local or regional research ethics review for interventions identified as research. The ECBA, however, will focus on the expected clinical benefits and risks for patients receiving interventions either in research or in clinical care in the early stages of development. Such review will require expertise and be independent of the clinicians who are involved in the development of the interventions. This suggests that the key challenge is to limit the introduction of emerging technologies to well-established research centers, which have sufficient expertise and a sufficient number of experts to make an independent assessment of the specific interventions being considered.

Our proposal is therefore that national governments should consider restricting the review and use in both research and clinical care of emerging technologies to specific, designated institutions. This would require legislative action, but there are already models for such legislation in countries such as Japan and China. Designated institutions would have the expertise necessary to provide a detailed and independent review of protocols in a way that is not possible in all local-level institutions. A possible model for this approach is the process in Japan for reviewing interventions that are classified as “highly difficult procedures”.

In response to several scandals related to innovative surgery procedures at some major hospitals around 2014 in Japan, the MHLW in 2016 introduced new regulations regarding “highly difficult surgical procedures”. This is a third scheme for the introduction of new surgical interventions, or the use of unapproved drugs, based on a revision of the enforcement regulations under the 1948 *Medical Care Act* (Ministry of Justice of Japan n.d.). In this scheme, a special category of interventions is defined as:

“Highly difficult and new medical technology which has not been used in the relevant hospital (excluding minor changes etc in operative methods) and that may result in death of or other serious impact on a patient” or “unapproved new pharmaceuticals […] Excluding those used for research”.

Interventions that satisfy this definition can only be provided in *Advanced Treatment Hospitals*. As of December 2022, this involved 88 hospitals, 80 of which are university hospitals. Based on the definition there are two types of such “highly difficult” interventions. They are either complicated, new surgical procedures or unapproved drugs. Both types of interventions need to be reviewed by specially designated committees, either the *Institutional Evaluating Committee of High Difficulty New Medical Technology* (for surgery) or the *Institutional Evaluating Committee of Unapproved New Drugs*, *and* *So* *Forth* (for drugs/devices/biologics) (Minamikawa et al. 2018). The rules require that justifications for the use are provided, in terms of benefits and risks of the proposed procedures compared with alternatives, as well as attention to informed consent requirements (Ministry of Health, Labour and Welfare of Japan n.d.). Although this was introduced mainly to deal with the review of surgical procedures, it could be broadly applicable to all advanced therapies, as they also often involve procedures in addition to drugs, and also to the new use of already existing drugs.

Japan has therefore introduced explicit national, procedural requirements for certain surgical procedures, as well as for unapproved drugs. In most other countries, only drugs are subject to formal, national evaluations before they can be used clinically. Most countries only allow already approved drugs for clinical use, but they can be used for unapproved, new therapeutic interventions, so-called off-label use. In Japan, at least in theory, their additional procedure for highly difficult interventions can also be used to clear drugs for specific clinical use, which have not yet been approved by national drug regulatory authorities. Many countries, including Japan, require additional approval for reimbursement by national health insurance schemes. Since 1980, in Japan, drugs can be reimbursed for unapproved use, if endorsed by professional organizations, or if there is ample scientific evidence for their effectiveness. Off-label use that has not yet been generally approved by professional organizations represents another challenge, typically discussed under the category of innovative treatment. Emerging technologies represent similar challenges: they often are applicable to few patients with serious conditions, and with few, existing options. In addition, they often do not just involve the administration of a drug but complex procedures as well. The Japanese scheme for local review of such procedures in designated institutions is therefore an interesting model.

The National Cancer Center Hospital in Japan has reported on their experience with an institutional mechanism for off-label use that falls outside the scope of the permitted use under the system introduced in 1980 but does appear to use a review mechanism similar to the one set up for approval under the “highly difficult care” system (Bun et al. 2021). Basically, a professional committee will review proposed cancer drugs for specific indications, which are generally approved, but not for the intended indication and have not yet been approved for reimbursement, which means that they are not recommended by professional organizations, nor is there overwhelming scientific evidence in their favor for that particular use. A high number of approved uses under this system are subsequently approved for reimbursement, which demonstrates that this can be a useful general procedure.

In Thailand, some medical schools have started recognizing this issue by clarifying and standardizing the pathway to apply medical innovation within the institute. For example, at the time of writing (2024), the Faculty of Medicine, Chulalongkorn University, was forming an ethical committee for advanced therapy and medical innovation and requires all applications of advanced therapy and medical innovation to get approval from this committee. Nevertheless, each institution has its own way of dealing with advanced therapy and medical innovation; some already see the necessity for this, although it is not yet a generally accepted procedure.

The main weakness of the current local, additional review systems is the potential lack of independence. The committees regulated by the Japanese *Medical Care Act* are institutional, and their independence is not legally required. Simply, the requirement of the composition of the former is that the committee shall involve at least the following three members: (1) a physician/dentist who belongs to the concerned clinical department, (2) a physician/dentist at any clinical department other than the concerned department, and (3) a physician/dentist belonging to the medical safety management division; and for the evaluation of drugs, it is required to involve (4) a pharmacist at the medical safety management division in addition to (1–3). Basically, they represent a return to the professional self-regulation model that existed before the introduction of the current requirements for independent research ethics review. One should be careful, however, not to overemphasize this as a problematic element. Effective review of clinical research and experimental care requires careful attention to benefit-risk assessment, the need for additional effectiveness and risk data before general adoption, and the scientific validity of the proposal, rather than detailed attention to informed consent requirements. Often, the appropriate experts for such assessments, also for the existing local ethics review committees, are scientists in the institution involved in the research or scientists in the same and other institutions who have a stake in what research is done. Nevertheless, there is a legitimate worry about the independence of committees largely comprised of experts in the field making professional judgments.

One way to alleviate this concern is to have a strong central oversight of the committee system with more specific requirements than currently in Japan for the composition of the committees and more specific registration and documentation requirements for the proceedings and decisions by these committees. It is also important to note that the requirement in many countries for additional review before reimbursement by national health insurance will also serve as a quality assurance of the local review, as documented by the experience at the National Cancer Center Hospital in Japan.

## Conclusion

In this paper, we have argued that there is a need to restrict advanced therapies, such as stem cell and gene therapies, to specific institutions, which have sufficient technical and ethics review capacities to review and provide such therapies responsibly. National governments need to introduce requirements for such institutions and review procedures, as well as oversight mechanisms. There is, however, no need to set up additional committees as this additional review can be based on existing local research ethics review committees, with the addition of specific expertise and procedures for the review of advanced therapies.
